# Combining Self-Organizing and Graph Neural Networks for Modeling Deformable Objects in Robotic Manipulation

**DOI:** 10.3389/frobt.2020.600584

**Published:** 2020-12-23

**Authors:** Angel J. Valencia, Pierre Payeur

**Affiliations:** School of Electrical Engineering and Computer Science, University of Ottawa, Ottawa, ON, Canada

**Keywords:** deformable objects, dynamic shape modeling, manipulation, robotics, shape, sensing

## Abstract

Modeling deformable objects is an important preliminary step for performing robotic manipulation tasks with more autonomy and dexterity. Currently, generalization capabilities in unstructured environments using analytical approaches are limited, mainly due to the lack of adaptation to changes in the object shape and properties. Therefore, this paper proposes the design and implementation of a data-driven approach, which combines machine learning techniques on graphs to estimate and predict the state and transition dynamics of deformable objects with initially undefined shape and material characteristics. The learned object model is trained using RGB-D sensor data and evaluated in terms of its ability to estimate the current state of the object shape, in addition to predicting future states with the goal to plan and support the manipulation actions of a robotic hand.

## 1. Introduction

In the context of robotic manipulation, object models are used to provide feedback signals that a robot can control when performing a specific task. For deformable objects, the object pose is not a sufficient state representation (Khalil et al., 2010) to guarantee even low-level manipulation tasks (e.g., pick-and-place), as manipulation actions produce changes in the object shape. Likewise, high-level manipulation tasks (e.g., making a bed or cleaning surfaces) involve knowledge of future behaviors to develop hierarchical plans. Therefore, an object model that integrates shape representation and prediction is required in order to perform a variety of tasks with deformable objects.

Early attempts to estimate the object shape in robotic manipulation mainly adopted an analytical approach, which is commonly adjusted in simulation (Nadon et al., 2018). This comes with some drawbacks in real robotic environments, as simulators are currently not sophisticated enough to provide realistic models of non-rigid objects (Billard and Kragic, 2019), and the support for sensor measurements and hardware in simulators is very limited. Furthermore, certain assumptions about objects are often made (e.g., homogeneous composition or isotropic materials). On the contrary, it is rarely possible to determine these conditions in advance for every new object encountered in the environment. This lack of a general-purpose methodology to estimate the object shape makes it difficult to develop more autonomous and dexterous robotic manipulation systems capable to handle deformable objects (Sanchez et al., 2018).

In this paper, we present a data-driven approach to estimate and predict the state of initially unknown deformable objects without the dependency on simulators or predefined material parameters. The contributions of this work can be summarized as follows: First, we develop a method for shape estimation using Self-Organizing Neural Networks (SONNs). Second, we design and implement an original method for shape prediction using Graph Neural Networks (GNNs) that leverages the initial SONN-based model. Third, we test the combination of the shape estimation and prediction methods as a learned model of deformable objects in real robotic environments. This paper represents a significant extension to previous work (Valencia et al., 2019) that corroborates the learned model across different types of deformable objects with experimental evaluations.

## 2. Related Work

Various methods that explore analytical modeling approaches for non-rigid objects in robotic environments are inspired by physics-based models, extensively studied in computer graphics (Nealen et al., 2006). These include continuous mesh models such as Euler-Bernoulli (EB) (Fugl et al., 2012), linear Finite Element Method (FEM) (Lang et al., 2002; Frank et al., 2014; Jia et al., 2014; Petit et al., 2015; Duenser et al., 2018) and non-linear FEM (Leizea et al., 2017; Sengupta et al., 2020). Also, discrete mesh models such as linear Mass-Spring Systems (MSS) (Leizea et al., 2014) and non-linear MSS (Zaidi et al., 2017) are considered. Additionally, discrete particle models such as Position Based Dynamics (PBD) (Güler et al., 2015) have been introduced. In these methods, a crucial step is to determine the material parameters of a deformable object (e.g., Young's modulus and Poisson's ratio). This is typically done via specific sensor measurements or assuming prior material information. More generally, these parameters are obtained by simultaneously tracking the shape while applying optimization techniques in the model.

Alternatively, data-driven approaches leverage sensor data to approximate the behavior of deformable objects typically using learning-based models. These include Single-layer Perceptron (SLP) (Cretu et al., 2012; Tawbe and Cretu, 2017). Other methods combine analytical and data-driven approaches in different parts of the modeling pipeline. For example, a Gaussian Process Regression (GPR) is used to estimate the deformability parameter of a PBD model (Caccamo et al., 2016). An Evolutionary Algorithm (EA) is proposed to search for the parameter space of an MSS model (Arriola-Rios and Wyatt, 2017). In these methods, an important aspect for a correct modeling is the information extracted from the sensor measurements. For RGB-D data, these correspond to properties of the shape (e.g., surfaces or feature points) and typically provide a structured representation suitable for the type of deformation model used. As such, B-spline snakes (Arriola-Rios and Wyatt, 2017) can be used to create a mesh-like representation. On the other hand, optical flow (Güler et al., 2015) and neural gas (Cretu et al., 2012) are used to create a particle-like representation.

Recent learning-based models such as Graph Neural Networks (GNN) have demonstrated the ability to act as a physics engine (Battaglia et al., 2016; Mrowca et al., 2018). Although there is little exploration of training such models using only sensor measurements. The most advanced attempt to model deformable objects beyond simulation is presented in Li et al. (2019a), where a real robotic gripper performs a shape control task on a deformable object. However, the models are initially trained entirely in simulation. Conversely, while aiming at exploiting real shape measurements for the modeling and prediction stages, this paper expands on the work of Cretu et al. (2012), as we aim to contribute a general-purpose methodology for modeling deformable objects in real robotic environments. In this way, we extend the latter by exploring recent learning-based models with physical reasoning capabilities (Battaglia et al., 2016) using RGB-D sensor measurements.

## 3. Methodology

**Figure 1:**
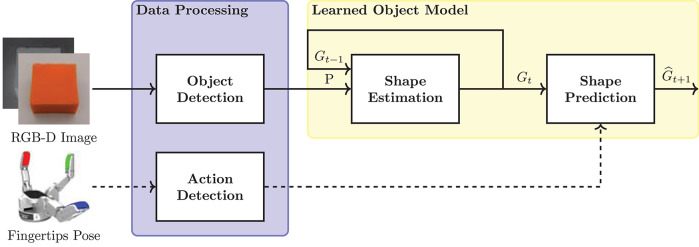
Components of the proposed data-driven approach for deformation modeling. The framework takes as input RGB-D sensor images and robotic hand's fingertips pose. (Data Processing) object and robot manipulation actions are detected. (Learned Object Model) the processed data is combined for the estimation and prediction of the object shape.

In this section, the proposed data-driven approach to model deformable objects is introduced (Figure 1). The main components of the learned object model are the shape estimation and prediction methods.

### 3.1. Shape Estimation

A Self-Organizing Neural Network (SONN) based model is proposed to estimate the object state from the sensor measurements. This model is called Batch Continual Growing Neural Gas (BC-GNG) and is an extension of the continual formulation of the Growing Neural Gas (C-GNG) algorithm (Orts-Escolano et al., 2015). C-GNG is extended by implementing a batch training procedure that enables to update the model parameters while avoiding an individual iteration on each sample during the execution of the algorithm. This approach provides benefits such as computational efficiency and faster convergence. First, the core principles of GNG models are described and then the technical details of our proposal are explained.

*Growing Neural Gas:* A GNG model (Fritzke, 1995) produces a graph representation *G* = (*O, R*) from a data distribution *P* of size *N*. Where, $O={\left\{{\text{o}}_{i}\right\}}_{i=1:N_{O}}$ is the set of nodes with $N_{O}$ cardinality, and $R={\left\{{\text{r}}_{k},u_{k},v_{k}\right\}}_{k=1:N_{R}}$ is the set of edges with $N_{R}$ cardinality, which connects an unordered pair of nodes *u*_*k*_ and *v*_*k*_. Also, each node has an associated feature vector **o**_*i*_ = {***x***_*i*_, *e*_*i*_}, which contains the position and spatial error, respectively. Likewise, each edge has an associated feature vector **e**_*k*_ = {*a*_*k*_}, which contains the connection age. The position is a direct measure of the spatial location of a node with respect to the sample, while the spatial error and connection age serve as measures for the addition and removal processes of nodes and edges from the graph.

The GNG model receives as input distribution, *P*, the current frame of the point cloud data associated to the object and produces the graph, *G*, as an estimation of the object shape. The model is trained following the execution of Algorithm 1. First, the graph is initialized by creating two nodes with position set to random values and spatial error set to zero. In addition, an edge connecting these nodes is created with age set to zero. After initialization, an individual sample, ξ, is randomly drawn from the distribution, and then the ADAPTATION and GROWING phases are run. During the former, nodes and edges features are sequentially updated, while during the latter and after receiving a certain number of samples, λ, new nodes and edges are added to the graph. These phases follow the original algorithm proposed by Fritzke (1995). In this work, the algorithm is executed until the quantization error (QE) reaches certain limit, which gives more flexibility to control the representation, as during the GROWING phase, nodes are dynamically created in an attempt to best fit the samples available in the input data, but does not require setting a fixed number of nodes. The quantization error is evaluated over the distribution *P* and computes the average difference between the closest node position (i.e., the smallest Euclidean distance) ***x***_*s*_1__ and the associated sample ξ.

(1) $$\begin{array}{r} \text{QE}=\frac{1}{N}\underset{\xi \in P}{\sum }||x_{s_{1}}-\xi || \end{array}$$

**Algorithm 1:** Steps of computation in GNG

**Input:** *P*
**Output:** *G*
1: *G* ← init_graph(*P*)
2: **while** QE > QE_max_ **do**
3: **for all** n ∈ *N* **do**
4: ξ ~ *P*
5: adaptation(*G*, ξ)
6: **if** (n **mod** λ) = 0 **then**
7: growing(*G*)
8: **end if**
9: **end for**
10: **end while**

*Outlier Regularization:* One problem that limits the use of GNG in problems with time constraints, such as tracking the shape of a deformable object as it evolves, relates to the requirement to retrain the model for every new input distribution collected by sensors. A continual formulation of the Growing Neural Gas (C-GNG) (Orts-Escolano et al., 2015) implements a technique that leverages the knowledge already learned during previous executions. Specifically, the graph from the previous data frame *G*_*t*−1_ is used to initialize the graph in the current data frame *G*. This provides a significant practical improvement but makes its formulation more sensitive. For example, outliers can affect the graph by creating nodes that do not adapt to the input distribution. The presence of these dead nodes represents a serious issue for the estimation of the object shape, especially when it is meant to vary over time. Therefore, we propose to regularize the influence of the outliers during the procedure that updates the position feature of each node. During the ADAPTATION phase, the nodes position are updated (Equation 2) for those that are found as closest ***x***_*s*_1__ or topological neighbors ***x***_*n*_ to the sample ξ. The parameters, ϵ_*s*_1__, and, ϵ_*n*_, correspond to the learning rates that control the influence of the adjustment of each contribution to the position feature.

(2) $$\begin{array}{r} \begin{array}{l} x_{s_{1}}\leftarrow x_{s_{1}}+\epsilon_{s_{1}}\left(\xi -x_{s_{1}}\right)\\ x_{n}\leftarrow x_{n}+\epsilon_{n}\left(\xi -x_{n}\right) \end{array} \end{array}$$

We introduce a new term, *w*_*s*_1__, that modifies the learning rate of the closest node position (Equation 3). In this way, those pairs of nodes and samples for which distances are large are penalized due to the possibility of being outliers, whereas those with small distances remain unchanged.

(3) $$\begin{array}{r} x_{s_{1}}\leftarrow x_{s_{1}}+w_{s_{1}}\epsilon_{s_{1}}\left(\xi -x_{s_{1}}\right) \end{array}$$

This regularization term (Equation 4) evaluates a 1D Gaussian kernel function with mean equal to the difference between the Euclidean distance ||***x***_*s*_1__−ξ|| and maximum quantization error QE_max_. And, standard deviation proportional to the maximum quantization error QE_max_.

(4) $$\begin{array}{r} w_{s_{1}}=\left\{ \begin{array}{cc} 1, & \mu <0\\ K\left(\mu ,\sigma \right)=e^{-\frac{\mu^{2}}{2\sigma^{2}}}, & \text{others} \end{array}\right. \end{array}$$

*Batch Training:* We also introduce a new procedure to update the features of the nodes and edges in batches, which unifies the contributions of a node with respect to its role among the samples. First, the node position is updated by combining the contributions when the node is found as closest and as topological neighbor. Similarly, the age of the edges connecting the closest node with its neighbors is updated by accumulating the times in which the node is found as closest. More specifically, the Euclidean distances between all the samples and nodes position are computed, also finding the two closest nodes at once. With this information, the input distribution can be represented as a set $P={\left\{P_{i}\right\}}_{i=1:N_{O}}$, where *P*_*i*_ is the batch data associated with each node found as closest, with size *N*_*i*_. In this way, the contribution of each node as closest is reformulated (Equation 5) as the average of the distances paired with that particular closest node.

(5) $$\begin{array}{r} x_{i}^{s_{1}}=\frac{1}{N_{i}}\underset{\xi \in P_{i}}{\sum }\left(\xi -x_{i}\right) \end{array}$$

Also, the age of the neighbor edges is reformulated as an increment of the batch data size *N*_*i*_. Since nodes are likely to be connected with more than one edge in the graph, the contribution of each node as neighbor requires an additional consideration. Initially, all the distances between the node and the samples associated due to the connections with all its neighbor nodes are collected, then the average of the collected distances is computed (Equation 6) similarly as in the previous step.

(6) $$\begin{array}{r} x_{i}^{n}=\underset{j}{\sum }\frac{1}{D_{i}N_{j}}\underset{\xi \in P_{j}}{\sum }\left(\xi -x_{i}\right) \end{array}$$

Where, *P*_*j*_ is the batch data of each neighbor of the closest node and *D*_*i*_ is the number of edges of the closest node. Thus, the contributions of each node as closest and as neighbor are included in a single expression to update the position feature (Equation 7), thus replacing the two-step update process with only an ADAPTATION phase in the online training, as detailed in Algorithm 2. By computing the Euclidean distance for all the samples at once, this procedure is also highly parallelizable as nodes can be updated independently.

(7) $$\begin{array}{r} x_{i}\leftarrow x_{i}+\epsilon_{s_{1}}x_{i}^{s_{1}}+\epsilon_{n}x_{i}^{n} \end{array}$$

**Algorithm 2:** Steps of computation in BC-GNG

**Input:** *P*, *G*_*t*−1_
**Output:** *G*_*t*_
1: **if** *t* = 1 **then**
2: *G* ← GNG(*P*)
3: **else**
4: *G* ← *G*_*t*−1_
5: **end if**
6: **while** QE > QE_max_ **do**
7: adaptation(*G, P*)
8: **end while**

### 3.2. Shape Prediction

As described in section 1, shape estimation alone does not provide sufficient information to perform high-level manipulation tasks. Therefore, a prediction phase must be incorporated in order to characterize the future states of a deformable object. With the objective to support the requirements of path planning and dynamic interaction of a robotic hand with a deformable object, Graph Neural Network (GNN) based models are also adapted in our framework to predict the future object state using the information of the current object state and the manipulation actions of the robotic hand. Specifically, we use the Interaction Network (IN) framework (Battaglia et al., 2016) along with its extension known as PropNet (Li et al., 2019b) for supervised learning on graph structures. Unlike standard GNNs, the IN is specifically designed to learn the dynamics of physical interactive systems. This model is characterized by being able to make predictions for future states of the system, and also to extract latent physical properties.

#### 3.2.1. Object-Action Representation

A new representation is created to jointly capture the object shape and the manipulation actions. This is defined as a directed graph *G* = 〈*O, R*〉. In which, $O={\left\{{\text{o}}_{i}\right\}}_{i=1:N_{O}}$ is the set of nodes with $N_{O}$ cardinality, and associated feature vector **o**_*i*_ = {***x***_*i*_, ***v***_*i*_}, which contains the object-action state defined as position and velocity. Also, $R={\left\{{\text{r}}_{k},v_{k},u_{k}\right\}}_{k=1:N_{R}}$ is the set of edges with $N_{R}$ cardinality, which due to the graph directionality connects an ordered pair of nodes, defined as sender node *u*_*k*_ and receiver node *v*_*k*_.

The object state is the shape estimation *G* produced by the BC-GNG model (section 3.1) and the manipulation actions are included as contact points, which are captured from the fingertips pose of the robotic hand. This means that new nodes are added to the graph with their feature corresponding to the position components of the fingertips pose. Also, edges are created when physical interactions are detected between the fingertips and the object, thus assigning action nodes for the fingertips as senders, and object nodes as receivers in the directed graph. The edge direction adds a causality property, indicating that action nodes produce the displacement of the object nodes and not the opposite. Furthermore, the velocity feature is computed by differentiating the signal obtained by the position feature of the object-action nodes.

#### 3.2.2. Interaction Networks

An IN model is trained to learn the transition dynamics of the object state. It takes the object-action graph at a certain time step *G*_*t*_ and outputs a prediction of the nodes position of the graph for the next time step ${\hat{G}}_{t+1}$. The model is updated following the execution of Algorithm 3, which uses the evaluation of aggregation and update functions (Gilmer et al., 2017) to perform computations with the graph features. The update function, ϕ_*R*_, is responsible to perform per-edge updates. This function evaluates the collected features of the edge along with the sender and receiver nodes, and thus computes the edge effect. Similarly, the update function, ϕ_*O*_, is responsible to perform per-node updates. This function evaluates the collected features of the node along with those produced by the update function, and thus computes the node effect. Since the update function produces a variable number of effects associated with each node, these are reduced using an aggregation function, ρ_*R* → *O*_, in order to produce a single effect.

**Algorithm 3:** Steps of computation in IN

**Input:** *G*_*t*_
**Output:** ${\hat{G}}_{t+1}$
1: ${\text{e}}_{k,t}\leftarrow \phi_{R}{\left({\text{r}}_{k,t},{\text{o}}_{u_{k,t}},{\text{o}}_{v_{k,t}}\right)}_{k=1:N_{R}}$
2: ${\text{e}}_{i,t}\leftarrow \rho_{R\to O}{\left({\text{e}}_{k,t}\right)}_{i=1:N_{O}}$
3: ${\hat{\text{o}}}_{i,t+1}\leftarrow \phi_{O}{\left({\text{e}}_{i,t},{\text{o}}_{i,t}\right)}_{i=1:N_{O}}$

The update functions are implemented as Multi-layer Perceptron (MLP) modules while the aggregation function is a summation. The mean squared error (MSE) of the predicted and observed nodes velocity (Equation 8) is used as the loss function to train the models. This statistical metric computes the average of the squared errors between the predicted velocities, ${\hat{v}}_{i,t+1}$, and the observed velocities, ***v***_*i, t*+1_.

(8) $$\begin{array}{r} \text{MSE}=\frac{1}{N_{O}}\overset{N_{O}}{\underset{i=1}{\sum }}{\left({\hat{v}}_{i,t+1}-v_{i,t+1}\right)}^{2} \end{array}$$

#### 3.2.3. Propagation Networks

A limitation of the IN occurs for systems that require long and fast propagation effects, since its formulation only considers local pairwise interactions during each time step. Therefore, several iterations of the algorithm are needed in order to propagate the information on the graph, and thus reach remote nodes. As an extension to IN, the PropNet (Li et al., 2019b) formulation (Algorithm 4) proposes the inclusion of a multi-step propagation phase, which consists of computing the edge and node effects using an additional iterative process, where *l* corresponds to the current propagation step parameter, and is set to a value within the range of 1 ≤ *l* ≤ *L*. Also, the update functions, $\phi_{R}^{\text{enc}}$, $\phi_{O}^{\text{enc}}$, are used to encode the input edge and node features, respectively. While the function, $\phi_{O}^{\text{dec}}$, is used to decode the output node feature. In this way, these functions learn a latent representation of the graph features, which are also part of the model during training.

**Algorithm 4:** Steps of computation in PropNet

**Input:** *G*_*t*_
**Output:** ${\hat{G}}_{t+1}$
1: ${\text{o}}_{i,t}^{\text{enc}}\leftarrow \phi_{O}^{\text{enc}}{\left({\text{o}}_{i,t}\right)}_{i=1:N_{O}}$
2: ${\text{r}}_{k,t}^{\text{enc}}\leftarrow \phi_{R}^{\text{enc}}{\left({\text{r}}_{k,t},{\text{o}}_{u_{k,t}},{\text{o}}_{v_{k,t}}\right)}_{k=1:N_{R}}$
3: ${\text{h}}_{i,t}^{0}\leftarrow 0$
4: **for all** *l* ∈ *L* **do**
5: ${\text{e}}_{k,t}^{l}\leftarrow \phi_{E}{\left({\text{r}}_{k,t}^{\text{enc}},{\text{h}}_{u_{k},t}^{l-1},{\text{h}}_{v_{k},t}^{l-1}\right)}_{k=1:N_{R}}$
6: ${\text{e}}_{i,t}^{l}\leftarrow \rho_{R\to O}{\left({\text{e}}_{k,t}^{l}\right)}_{i=1:N_{O}}$
7: ${\text{h}}_{i}^{l}\leftarrow \phi_{O}{\left({\text{o}}_{i,t}^{\text{enc}},{\text{e}}_{i,t}^{l},{\text{h}}_{i,t}^{l-1}\right)}_{i=1:N_{O}}$
8: **end for**
9: ${\hat{\text{o}}}_{i,t+1}\leftarrow \phi_{O}^{\text{dec}}{\left({\text{h}}_{i,t}^{L}\right)}_{i=1:N_{O}}$

## 4. Experimental Evaluation

### 4.1. Experimental Setup

**Figure 2:**
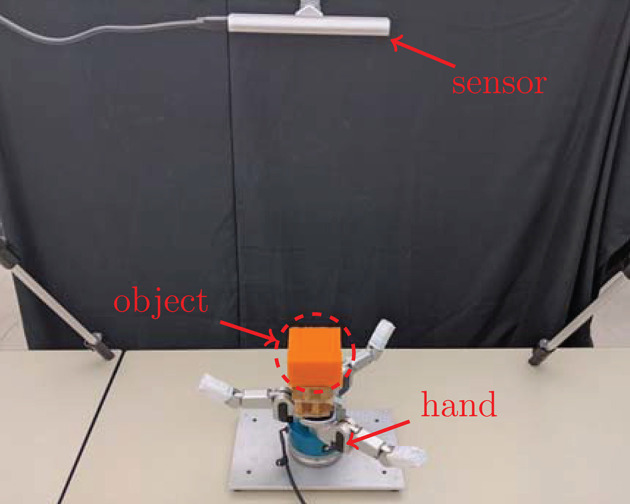
Configuration of the real robotic environment. The location of the RGB-D sensor, deformable object and three-fingered robotic hand are marked in red.

**Figure 3:**
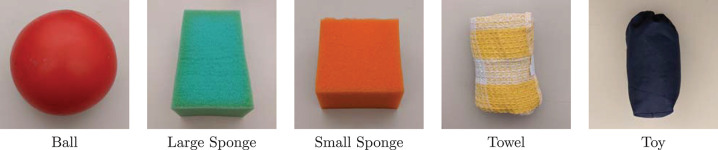
Deformable objects used to construct the dataset. (Ball) plastic type found in toy stores. (Sponges) foam type used as cleaning utensils. (Towel) textile type found in warehouses. (Toy) stuffed type found in pillows or stuffed animals.

The configuration of the real robotic environment is shown in Figure 2, which consists of a Barrett BH8-280 robotic hand^1^ resting on a flat table, an Intel RealSense SR305 RGB-D sensor^2^ mounted overhead on a tripod, and a deformable object placed on the palm of the robotic hand. The complete set of deformable objects used to construct the datasets is shown in Figure 3.

All the sensors and hardware components used in the robotic manipulation setup are operated through ROS (Quigley et al., 2009). The data preparation, signal and image processing steps are implemented using SciPy (Virtanen et al., 2020) and OpenCV (Bradski, 2000) libraries. The models are implemented in the Deep Graph Library (Wang et al., 2019) using PyTorch (Paszke et al., 2019) as backend.

### 4.2. Sensor Measurements and Data Processing

The RGB-D sensor data is processed in a ROS node to detect the object and generate the point cloud data. Also, another ROS node is used to estimate the robotic hand's fingertips pose to generate the manipulation action information.

#### 4.2.1. Object Detection

Classical image segmentation techniques are applied to both aligned color and depth images for the detection of the deformable objects. The color image is transformed to the HSV color space, and then a histogram backprojection technique is applied to obtain a binary mask. Then, the mask is filtered by applying a convolution with threshold operation to obtain a cleaner result. Moreover, the depth image is cropped by volume, truncating the spatial values based on available information about the object position relative to the camera. Thus, the resulting color and depth masks are combined and applied to the depth image to obtain the object of interest. The segmented image is then deprojected to convert the 2D pixels to 3D point clouds. For the small sponge object, this process transforms the RGB-D sensor images from 640 × 480 to approximate 80 × 80 × 3.

#### 4.2.2. Fingertips Pose Estimation

The data captured on the fingertips correspond to the pose (position and orientation) of each tip. To facilitate the accurate estimation of the pose, a set of AR markers are placed on each tip. The design is based on the ARTags fiducial marker system, and generated according to the following parameters: size of 1.8 cm, margin of 1-bit, and pattern of 25-bit 5 × 5 array. The latter controls the number of tags that can be created based on the marker dictionary. Given the physical dimensions of the robotic hand, this design enables to precisely fit each marker on the tip. In turn, the markers are visible enough to be detected in the images captured by the RGB-D sensor. The fingertips pose corresponds to that estimated by the markers. The pose enables to define the contact points, which is determined by a contact region with spherical shape, centered on the marker and with a radius of 2.3 cm. The latter is measured considering the tip size relative to the marker location.

### 4.3. Shape and Motion Estimation With GNG-Based Models

These experiments are run on a computer with 1 × Intel Core i5-7300U @ 2.60 GHz, 16 GB RAM, and GNU/Linux operating system. The parameters of the GNG models are shared as much as possible in order to consistently compare the performance of the different variations. For GNG, an age of 35, learning rate of 0.1 and 0.005, error decay of 0.5 and 0.9995 are used. For C-GNG, an age of 2,000, learning rate of 0.1 and 0.005. And for BC-GNG, an age of 2,000, learning rate of 0.4 and 0.01 are used. The sigma value of the regularization term used in C-GNG and BC-GNG corresponds to 0.6 for the towel and 4 for the rest of the objects.

**Figure 4:**
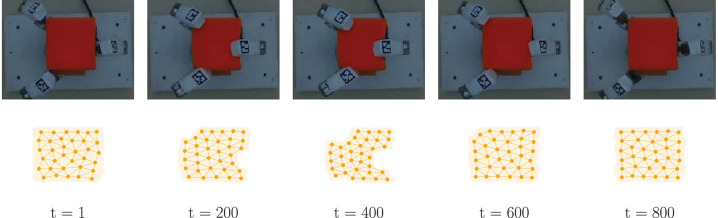
Qualitative results of the shape estimation method. **(Top)** color image sequence of the scene with the small sponge at different deformation levels. **(Bottom)** point cloud and graph representation of the object shape obtained by the BC-GNG model.

The fingers trajectory are generated to perform a squeeze-like manipulation with each object. The base joints range is limited to (−90°,90°), whereas the spread joint is limited to (−45°,45°). Each trajectory is generated taking as final configuration a random joint position within the available moving range for each robotic finger, and using a linear interpolation with 50 points beginning from a predefined rest position of the hand. The trajectories are designed in this manner to produce brief rest periods at the end of each point with the intention of preventing slippage or sliding movements of the object, and thus mainly capturing information associated with the deformation. A dataset is created which consists of a file with 800 samples, using a sampling rate of 30 Hz. Each file stores the data generated in synchronization with the execution of the fingers trajectory, which takes approximately 27 s to complete. Results for a subset of the data frames that progressively reflects various deformation levels using the small sponge as an example of deformable object are shown in Figure 4. We refer the reader to the Supplementary Material for additional results with the other deformables objects considered, as per Figure 3.

As mentioned in section 2, the properties extracted from the object shape are the basis for any learned model. This means that motion changes should closely capture the dynamics of the deformation. A motion analysis can be used to determine whether the produced shape estimation is consistent with the deformation and reflects the current state of the deformable object. The latter is formalized by also considering the requirements of real robotic environments.

#### 4.3.1. Real-Time Execution

**Figure 5:**
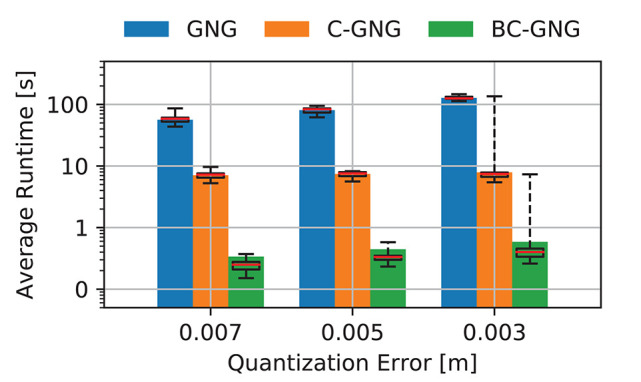
Average of runtime sequence. GNG models formation computing time with respect to varying tolerance on model accuracy during the manipulation of the small sponge object.

We evaluate the performance of different variations of GNG for real-time shape estimation using point clouds as input data. The runtime of each model is recorded per data frame and the average over the entire manipulation trajectory is computed, as shown in a bar plot in Figure 5. The models are evaluated using three levels of quantization error, which are selected to provide an insight of the precision costs associated to the representation. For GNG, the runtime takes an average of 80.7 s when a quantization error tolerance of QE = 0.005 is imposed, and grows linearly if more precision (lower quantization error) on the shape representation is required. On the other hand, C-GNG runtime is several orders of magnitude faster mainly due to the reuse of the previous graphs over iterations. Although, this formulation is a great improvement, its runtime is not yet suited for real-time applications, at least for low-power CPUs and embedded systems. It takes an average of 7.4 s at each data frame but reveals less sensitive to the tolerance set on the model precision. Finally, the proposed BC-GNG variation that involves batch training considerably speeds up the execution. In this case, the algorithm needs an average of 0.4 s to construct the same graph with only a slight variation in computing time when the desired model accuracy is varied. In certain cases, sudden increase in time is observed when more accuracy is required, as shown in QE = 0.003. This occurs in data frames with high variations, since graphs with a fixed number of nodes cannot always adapt to such levels of accuracy. Therefore, early stopping mechanisms are required to avoid unnecessary iterations.

#### 4.3.2. Temporal Smoothing

**Figure 6:**
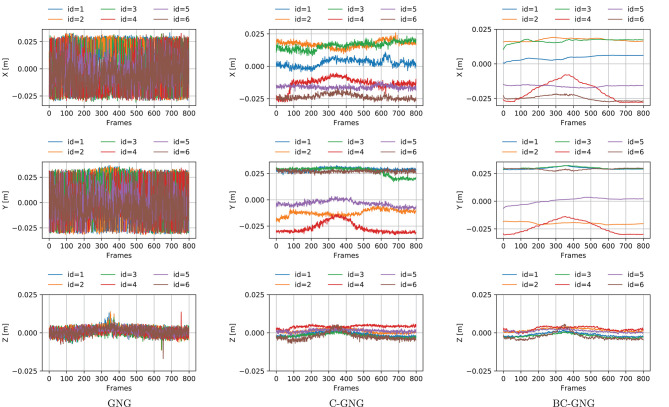
Local displacements of nodes encoding the object shape. Three-dimensional coordinates of nodes obtained by the GNG models during the manipulation of the small sponge object.

We evaluate the performance of different variations of GNG to generate stable displacements of the nodes that encode the object shape. The path followed by each individual node is measured relative to the centroid coordinate system to mitigate the influence of rigid motions. These local displacements estimate the actual deformation motion of the object shape. The 3-dimensional temporal evolution of local nodes for the small sponge object is shown in Figure 6 for a subset of nodes (first 6 out of 34 nodes) extracted from the graph forming the shape model and over the 800 frames that correspond to a manipulation operation.

The continual models (C-GNG and BC-GNG) clearly produce more stable signals. An interesting property of BC-GNG is the low-pass filter effect that is observed in the signals. This behavior occurs due to the characteristic of the algorithm that uses the average of the nodes position during the update process. Therefore, the node displacements obtained by BC-GNG are much smoother, and desirable to estimate with confidence the motion and deformation quantities of a non-rigid object as its shape is not dominated by noise associated with the individual dynamics of nodes forming the graph-based representation.

#### 4.3.3. Region Correspondence

Finally, we evaluate the performance of different variations of GNG to produce node displacements (Figure 6) that can be used as features of the object motion. The region correspondence of nodes position is non-existent in GNG due to the stochastic nature of the algorithm, which causes that new nodes are not created around the same location. For C-GNG, the displacements exhibit a localized motion of nodes position that preserve certain regions of the shape. However, there still exists some interference between nodes which causes unrecoverable positions and affects the region correspondence of the representation, more noticeable when large deformations occur. For BC-GNG, these displacements reflect a more localized motion of nodes position, even further to that observed in C-GNG. Interference between nodes is not causing strong deviations in their displacements, hence better preserving their correspondence throughout the manipulation task.

### 4.4. Deformation Dynamics Prediction With GNN-Based Models

These experiments are run on a cloud instance with 1 × Intel Xeon Processor @ 2.3GHz, 1 × NVIDIA Tesla K80 GPU with 2,496 CUDA cores, 12 GB RAM GDDR5 VRAM, and GNU/Linux operating system. The training procedure of the GNN models consists of 20 iterations, and using a batch size of 1. The MLP modules are trained using the Adam optimizer (Kingma and Ba, 2015) with learning rate of 0.001 and momentums of 0.9 and 0.9999. A learning rate scheduler with factor of 0.8 and patience 3 is used.

The architecture design of the GNN models follows the configuration presented in Li et al. (2019a). This configuration is shared among models in order to consistently compare their performances, hence the main difference is the propagation step parameter L, which is 1 for IN and 2 for PropNet. In this way, the encoder functions $\phi_{R}^{\text{enc}}$, $\phi_{O}^{\text{enc}}$ are 2-layer MLPs with hidden sizes of 200 and 300, with output size of 200. The update functions ϕ_*R*_, ϕ_*O*_ are 1-layer MLPs with hidden size of 200, and output size of 200. And, the decoder function $\phi_{O}^{\text{dec}}$ is 2-layer MLP with hidden size of 200 and output size of 3, the latter corresponds to the components of the predicted velocity. All MLP modules use the rectified linear unit (ReLU) as the activation function. A dataset of graphs per object is created and consists of 20 files, each associated to a different fingers trajectory. This produces 16,000 samples in total. The dataset is divided into 80% for training, 10% for validation and 10% for test, which is equivalent to 16 trajectories (12,800 randomly shuffled samples) and 2 × 2 trajectories (2 × 1,600 samples) respectively. In addition, the dataset is normalized between 0 and 1 due to the varied scales of the position and velocity features.

The GNN models are primarily analyzed in two situations: first evaluating the performance of the predictions for the object deformation in single-step time sequences, and then evaluating the ability to generalize over multi-step time sequences. In order to enable a more direct interpretation of the results, the Root Mean Square Error (RMSE) of the predicted and observed nodes position is used as a metric. Thus, the nodes position of the next frame ${\hat{x}}_{i,t+1}$ are calculated via explicit integration of the equation of motion (Equation 9), which uses the predicted velocities of the next frame ${\hat{v}}_{i,t+1}$ and time per frame Δ*t* to update the current position of each node.

(9) $$\begin{array}{r} {\hat{x}}_{i,t+1}=x_{i,t}+\Delta t\cdot {\hat{v}}_{i,t+1} \end{array}$$

**Figure 7:**
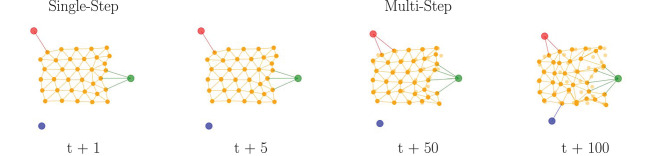
Qualitative results of the shape prediction method. Graph sequences predicted by the GNN model (PropNet) over different time horizons for the small sponge with three contact points (nodes in red-green-blue). The predicted shape corresponds to the nodes position produced by the model. The observed nodes position are displayed in shaded color.

*Single-Step Predictions:* The nodes position are predicted from the most recent observed data at each frame (t + 1). The GNN models obtain a relatively low and consistent error (Table 1) of the nodes position throughout the entire range of acquired data frames over the object manipulation duration. These results confirm a stable prediction capability, one step ahead, with the GNN models, as shown on the left of Figure 7.

**Table 1:** Prediction error of nodes position.

**GNN models**	**Frame steps**		
	**t + 1**	**t + 5**	**t + 50**
IN	0.08 ± 0.02	9.52 ± 7.14	46.28 ± 30.53
PropNet	0.08 ± 0.02	9.53 ± 7.14	53.66 ± 37.08

*RMSE (10^-5^) values in meters obtained by the GNN models at different time steps during the manipulation of the small sponge*.

*Multi-Step Predictions:* The nodes position are predicted for every frame but with updates from observed data fed into the model at different frames (t > 1), which involves a longer-term prediction before new data is made available to the GNN models. The error produced by the models remains relatively low (Table 1) over a short range of frames (t + 5), but progressively degrades as the number of frames further increases (t + 50). As a consequence, at some point the models become unable to predict with confidence the nodes position, as shown on the right of Figure 7. The errors from previous iterations cumulate and the prediction diverges, causing the deformable object prediction to enter an unrecoverable state.

## 5. Discussion

### 5.1. Shape Estimation Quality

All variants of GNG studied in this research produce a graph sequence that estimates the object shape. However, regardless of maintaining consistency during model training (i.e., shared parameters and stopping criterion), the proposed BC-GNG model performs better in terms of computing time and motion estimation, as demonstrated experimentally in section 4.3. Consider the data frames (Figure 4) where the largest deformation occurs (around *t* = 400). The areas on the shape where the object is compressed more (e.g., around the center and vertices) show a higher and more natural accumulation of nodes. Also, the estimation obtained when no interaction occurs between the fingers and the small sponge (around *t* = 800) produces a more symmetric node density that better resembles the object topology. These characteristics are also observed in the other deformable objects considered in these experiments.

We also observed that BC-GNG still exhibits some difficulty to recover the initial node position for elastic objects. Unlike C-GNG, such variations do not manifest as abrupt changes in the signal due to the smoother characteristic of the displacements. This behavior is more desirable since abrupt changes are directly associated with large deformations, which on the contrary do not correspond to the reality of what the object is experiencing. In particular, local displacements of large volumetric objects are more affected. These might be related to occlusions causing correspondence problems by further reducing the amount of points reported by the sensor when the object is manipulated.

### 5.2. Shape Prediction Reliability

The main advantage of combining a GNN predictive model (IN and PropNet) with a self-organizing model (BC-GNG) is the fact that the training data generated by the latter are dynamic graphs with efficient size. As noted in Li et al. (2019a), training GNNs with large static graphs may overload memory capacity and delay convergence. Furthermore, such models do not perform well in dynamical settings due to unnecessary interactions associated to a fully connected graph topology. The proposed combination of models contributes to overcome these important constraints, which can be detrimental to successful robotic manipulation of deformable objects. Thus, the GNN models trained in combination with the BC-GNG graphs effectively capture the immediate changes of the object shape when evaluated in single-step, or short-term, time sequences and demonstrate potential to produce robust and visually plausible predictions of the deformation dynamics. On the other hand, while their performance tends to degrade over longer term predictions, anticipating an object's shape deformation a few steps ahead is representative of what human beings can realistically achieve, and generally proves sufficient for robotic manipulation supported by modern RGB-D sensors that can now capture point clouds in real-time. Given that the modeling and prediction framework is meant to be part of the robotic hand control loop, new RGB-D data is made available to update the deformable object representation, and provide an updated prediction, at the same frame rate as the robot controller. As a result, long-term prediction is not of essence in this type of application. According to the configuration used, we also notice that the performance of the GNN models are very similar. Although, the latter could be affected by the fact that PropNet shows faster convergence in training than IN due to the multi-step propagation phase.

## 6. Conclusion

This paper presents a first attempt at using graph models to learn the dynamics of deformable objects entirely from RGB-D sensor measurements. The proposed BC-GNG formulation improves the performance over C-GNG by producing graphs with better node stability, correspondence in regions with shape variations and lower computational cost. These properties enable to combine other graph models such as GNNs to predict the deformation dynamics of non-rigid objects.

By combining the relational structure of self-organizing and graph neural networks, the proposed approach successfully captures the object shape and predicts the deformation dynamics when evaluated over single-step or short-term time sequences. In comparison to analytical models, execution time is faster and information on the shape and physical properties of the object does not need to be known or approximated a priori. Therefore, the proposed combination of graph models and their adaptation demonstrate strong potential for characterizing deformable objects' shape and dynamics, as required to support advanced dexterous robotic manipulation.

## Data Availability Statement

The raw data supporting the conclusions of this article will be made available by the authors, without undue reservation.

## Author Contributions

Both authors contributed to the overall conception of the methodology, experimentation, analysis and manuscript writing.

## Conflict of Interest

The authors declare that the research was conducted in the absence of any commercial or financial relationships that could be construed as a potential conflict of interest.

## Footnotes

^1^https://advanced.barrett.com/barretthand

^2^https://www.intelrealsense.com/depth-camera-sr305
