# Supplementary material for: Combining Self-Organizing and Graph Neural Networks for Modeling Deformable Objects in Robotic Manipulation
Source: Front Robot AI. 2020 Dec 23;7:600584. doi: 10.3389/frobt.2020.600584 (PMC7806087; doi:10.3389/frobt.2020.600584)
Supplement: Supplementary file 1 [file Data_Sheet_1.PDF]

Supplementary Material

1 QUALITATIVE EXAMPLES

Qualitative results for four additional deformable objects are shown in Figures S1 to S7.

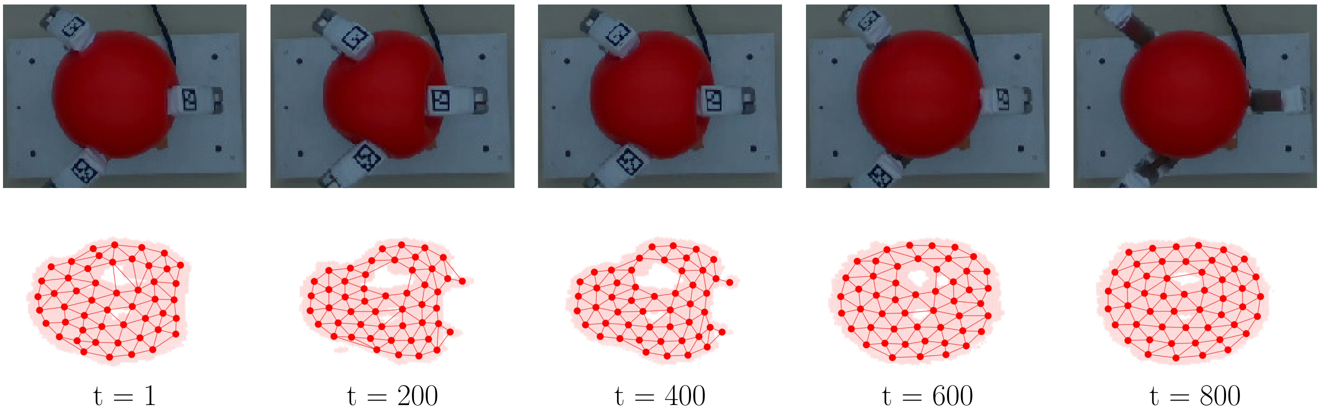

Figure S1. Qualitative results of the shape estimation method for the ball object. (Top) color image sequence of the scene at different deformation levels. (Down) point clouds and graph sequences obtained by the BC-GNG model.

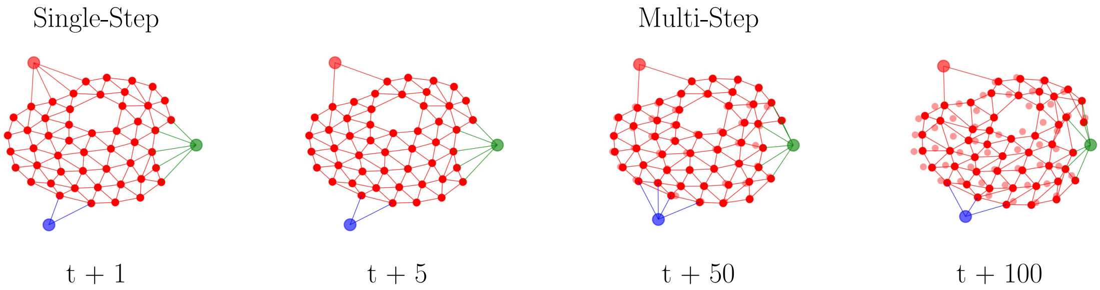

Figure S2. Qualitative results of the shape prediction method for the ball object. Graph sequences predicted by the PropNet model at different time horizons.

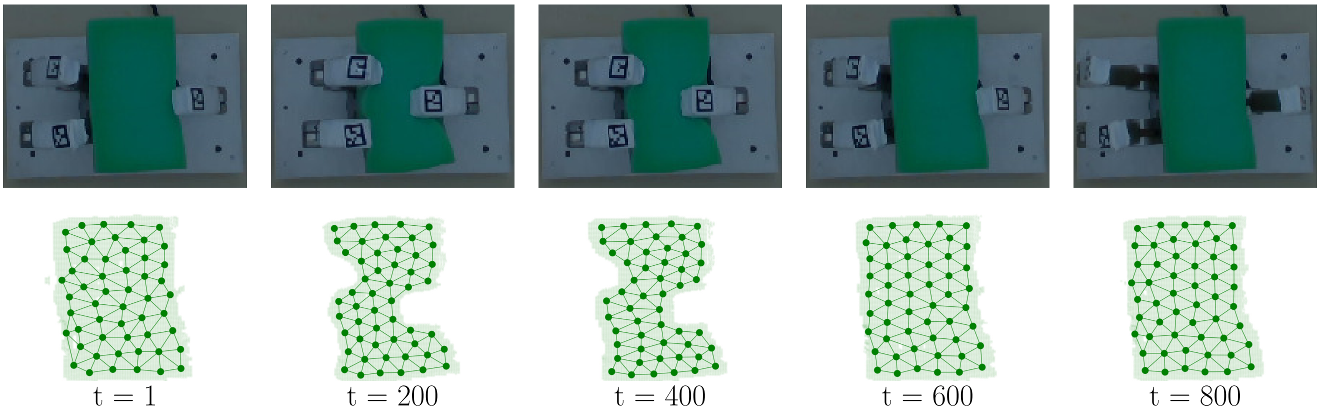

Figure S3. Qualitative results of the shape estimation method for the large sponge object. (Top) color image sequence of the scene at different deformation levels. (Down) point clouds and graph sequences obtained by the BC-GNG model.

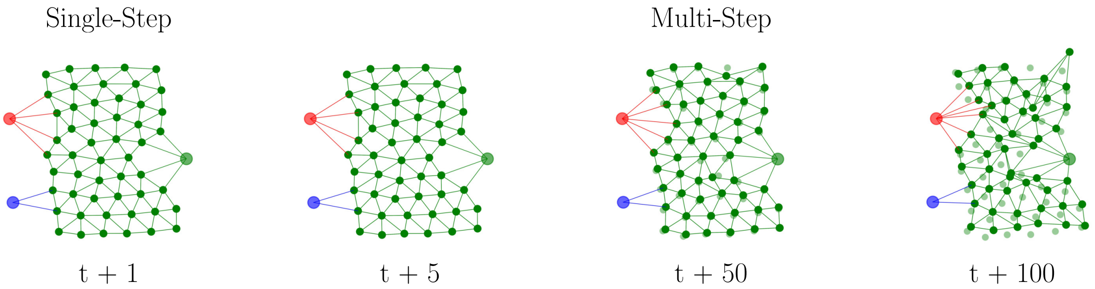

Figure S4. Qualitative results of the shape prediction method for the large sponge object. Graph sequences predicted by the PropNet model at different time horizons.

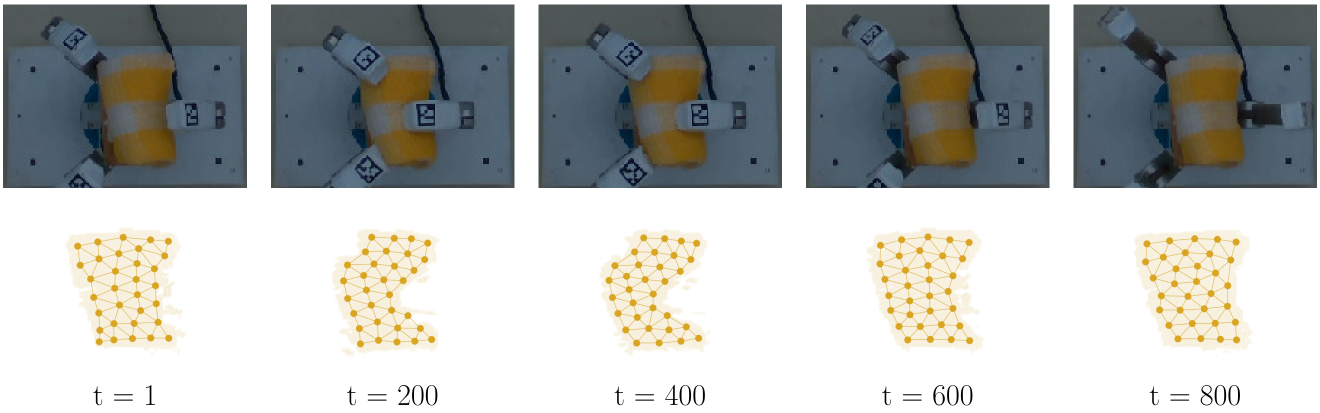

Figure S5. Qualitative results of the shape estimation method for the towel object. (Top) color image sequence of the scene at different deformation levels. (Down) point clouds and graph sequences obtained by the BC-GNG model.

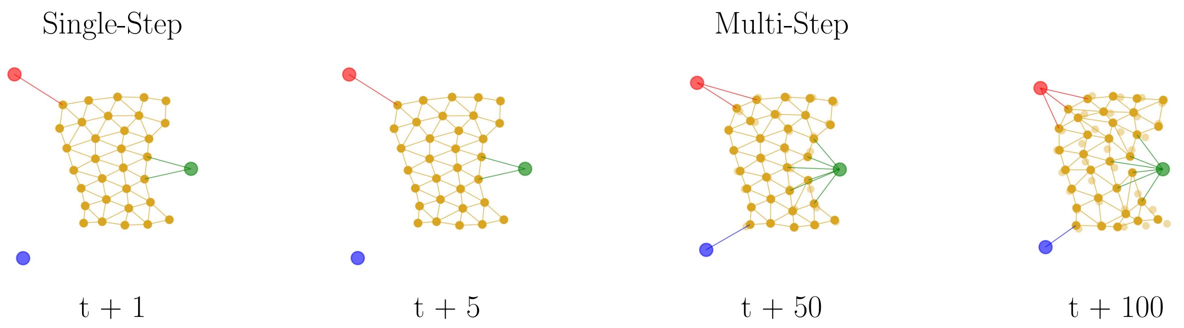

Figure S6. Qualitative results of the shape prediction method for the towel object. Graph sequences predicted by the PropNet model at different time horizons.

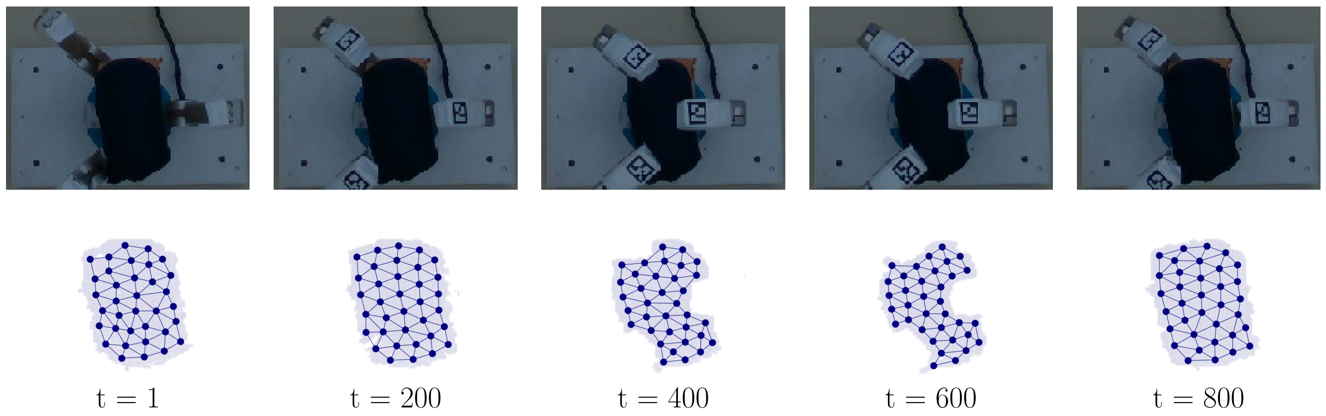

Figure S7. Qualitative results of the shape estimation method for the toy object. (Top) color image sequence of the scene at different deformation levels. (Down) point clouds and graph sequences obtained by the BC-GNG model.

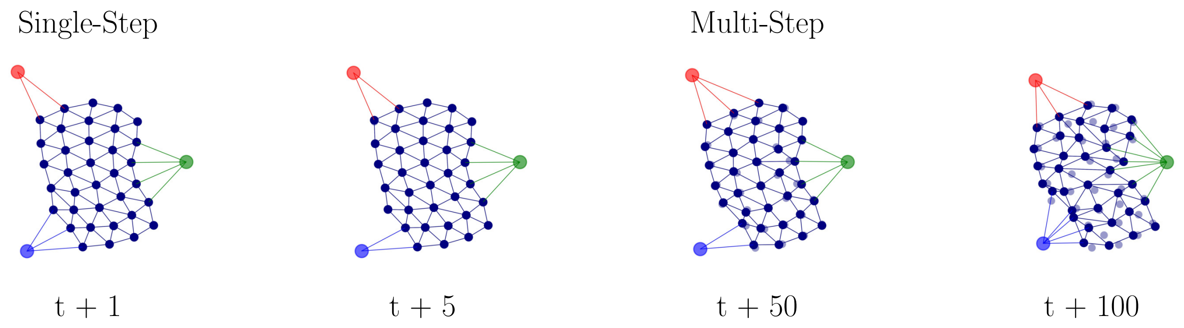

Figure S8. Qualitative results of the shape prediction method for the toy object. Graph sequences predicted by the PropNet model at different time horizons.
